# mSumireF a Monomeric Violet Fluorescent Protein

**DOI:** 10.1021/acsbiomedchemau.5c00175

**Published:** 2026-01-06

**Authors:** Jacob Kress, Sookyeong Kim, Caitlynn Bryant, Emily Camila Lopez-Lopez, Jiali Zha, Mathew Tantama

**Affiliations:** Department of Chemistry & Biochemistry Program, 8456 Wellesley College, 106 Central Street, Wellesley, Massachusetts 02481, United States

**Keywords:** monomeric violet fluorescent protein, Sumire, oligomerization, hetero-FRET donor, homo-FRET

## Abstract

The development of genetically encoded labels for multicolor experiments requires a diverse palette of fluorescent proteins that are well-behaved. Here, we report mSumireF, a monomeric variant of the violet fluorescent protein Sumire. On its own, the canonical monomerizing valine-to-lysine mutation at residue 206 causes a significant loss of brightness for the mSumire variant. We found that brightness is recovered upon the reversion of mSumire’s tyrosine at position 165 back to phenylalanine as in its superfolder GFP grandparent. Importantly, this mSumireF variant exhibits significantly less oligomerization tendency in the organized smooth endoplasmic reticulum (OSER) assay in mammalian cells and in protein solution assays. Furthermore, we demonstrate that an mSumireF donor can be effectively paired with the fluorescent protein acceptors mCerulean3, mTurquoise2, and LSSmScarlet to generate FRET-based ATP biosensors. mSumireF thus provides an improved violet fluorescent protein to expand color options with reduced concern of unwanted dimerization.

## Graphical abstract

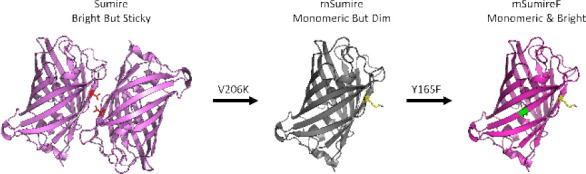

## Introduction

A challenge of any multiplexed fluorescence experiment is to find spectrally compatible labels or biosensors. To this end, a great deal of progress has been made in the expansion of the fluorescent protein color palette.^1^ Many of the currently available fluorescent proteins have also been evolved for desirable properties beyond their color, including increased brightness, decreased photobleaching, altered pH sensitivity, and reduced oligomerization.^2–7^ In particular, monomerization continues to be a priority as new fluorescent proteins are developed because unwanted oligomerization can cause biological artifacts and toxicity when expressed in cells.^7–11^

In regards to the current fluorescent protein color palette, there are still few options toward the violet end of the visible spectrum. Recently, Sugiura and Nagai engineered a bright violet fluorescent protein called Sumire that is nearly four times brighter than Sirius.^2,12^ However, Sumire is based on superfolder green fluorescent protein (sfGFP), which still has a propensity to dimerize.^7,13^ We therefore generated and characterized a monomeric variant, mSumireF, that has similar brightness but a significantly reduced tendency to oligomerize (Figure 1).

**Figure 1:**
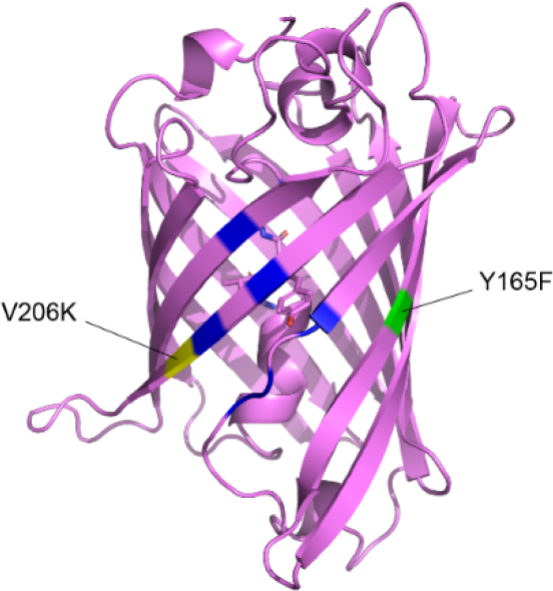
mSumireF. The sfGFP (PDB: 2B3P) structure is shown in pink. Blue highlights residues mutated to generate Sumire. Yellow and green highlight locations of the mutations to increase monomeric character and brightness, respectively.

## Methods

### Molecular Biology

Sumire-pRsetB was a gift from Takeharu Nagai (Addgene plasmid# 193361; http://n2t.net/addgene:193361; RRID:Addgene_193361). V206K and Y165F mutations were introduced by overlap-extension PCR site-directed mutagenesis. pCytERM_mScarlet_N1 was a gift from Dorus Gadella (Addgene plasmid# 85066; http://n2t.net/addgene:85066; RRID:Addgene_85066). Sumire variants were cloned into the pCytERM backbone by NEB HiFi cloning to generate the p450 fusions for the OSER assay. pDx_mScarlet-I3 was a gift from Dorus Gadella (Addgene plasmid# 189757; http://n2t.net/addgene:189757; RRID: Addgene_189757). mScarlet-I3 fusions to each Sumire variant were generated by NEB HiFi cloning within the pRset backbone. pENTR-p38KTRmCerulean3 was a gift from Markus Covert (Addgene plasmid# 59149; http://n2t.net/addgene:59149; RRID:Addgene_59149). mTurquoise2-N1 was a kind gift from Richard Day. DsRed-ER was a kind gift from Louise Darling. The LSSmScarlet gene was synthesized as a gBlock (IDT). ATP sensors were constructed by NEB HiFi cloning in the pRset backbone using the *B. subtilis* epsilon subunit from a previously published sensor (GW1-ARSeNL; Addgene plasmid# 207873; http://n2t.net/addgene:207873 ; RRID:Addgene_207873).^14^

### Protein Characterization

Sumire variants were expressed in BL21­(DE3) *E. coli* ^2^ and purified by nickel-affinity fast protein liquid chromatography. Protein concentrations were determined by Bradford assay, and extinction coefficients were determined by Beer–Lambert plots. Fluorescence quantum yields of the Sumire variants were determined using the slope of fluorescence intensity versus absorbance relative to that of wildtype Sumire, using its reported quantum yield.^2^ Data were collected in the linear range with absorbances less than 0.1 to minimize inner filter effects (Figure S1). Fluorescence anisotropy oligomerization assays were conducted on a Jasco FP-8350 spectrofluorometer with automated g-factor determination. All other fluorescence assays were carried out on a Molecular Devices SpectraMax ID5 microplate reader. ATP dose response curves were collected using 0.5 μM protein. Origin 2025 (OriginLab) was used for nonlinear curve fitting.

### Microscopy

HEK293 cells were maintained in low glucose Dulbecco’s Modified Eagle Media supplemented with 10% Cosmic Calf Serum. Cells were separately electroporated with CytERM-Sumire^7^ or CytERM-mSumireF mammalian expression plasmids using a Mirius EZporator and plated onto #1.5 18 mm glass coverslips. Two days post-transfection, cells were fixed with 4% paraformaldehyde, blocked with 3% bovine serum albumin and permeabilized with 0.1% Triton-X100 in phosphate buffered saline prior to staining with rabbit anti-GFP primary antibody (Abcam ab290) and goat antirabbit AlexaFluor488 secondary antibody (Invitrogen A-11008). Images were taken with a Leica TCS SP5 laser scanning confocal microscope using a 63X oil immersion objective with argon laser excitation at 488 nm and the green emission channel from 500 to 530 nm. For live-cell widefield fluorescence microscopy, cells were imaged in Dulbecco’s phosphate buffered saline including magnesium and calcium and supplemented with glucose. Violet fluorescence was collected with a Nikon ET-DAPI (96360) cube, and red fluorescence was collected with a Nikon DsRed (96364) cube. Live-cell imaging was carried out on a Nikon Ti–U inverted microscope with a metal halide lamp, QI Click monochrome CCD camera, and 40X air objective.

### Live Cell Spectroscopy

T7 Express lysY/Iq *E. coli* (NEB) were transformed with separate vectors each coding for mScarlet-I3 ^15^ fused to a Sumire mutant or transformed with pUC19 as a nonfluorescent control. Luria broth cultures with carbenicillin selection were inoculated with single colonies. Cells were grown to an OD600 of approximately 0.6 at which point protein expression was induced with 1 mM IPTG. Starting approximately 60–90 min after induction, spectra were collected every 30–60 min. Two spectra were collected for each time point, one with direct excitation of Sumire at 340 nm and one with direct excitation of mScarlet-I3 at 530 nm. Fluorescence emission intensities were measured at 410 and 592 nm, respectively. To account for background autofluorescence, fluorescence emission intensities from the pUC19 control cells were subtracted from the Sumire and mScarlet-I3 intensities. Corrected fluorescence emission intensities were then plotted with respect to time.

## Results and Discussion

### Photophysical Characterization

Residue 206 at the GFP dimer interface is commonly mutated to monomerize its derivatives, and we first attempted to install the V206K msfGFP mutation to monomerize Sumire (Figure 1).^7,16^ Unfortunately, the resulting mSumire variant shows a 4-fold lower brightness caused by significant decreases in both the extinction coefficient and quantum yield (Table 1 and Figure S1). In order to recover brightness, we explored additional mutations to mSumire.

**Table 1:** Photophysical Properties

	ε^a^	φ^b^	Brightness^c^	K_D,app_ ^d^
Sumire	18600 ± 100	0.70^e^	13 (100%)	2.4 ± 0.4
mSumire	9060 ± 20	0.32 ± 0.01	3 (20%)	
SumireF	23590 ± 70	0.71 ± 0.02	17 (130%)	3.3 ± 0.3
mSumireF	14700 ± 40	0.54 ± 0.01	8 (60%)	≥80
Sirius^f^	15,000	0.24	4 (30%)	

a Extinction coefficient in M^–1^·cm^–1^.

b Fluorescence quantum yield.

c Brightness = ε x φ; percent of Sumire parent in parentheses.

d Apparent self-affinity binding dissociation constant in μM.

e Data from ref. 2

f Data from ref. 12

Serendipitously, we found the Y165F mutation recovers brightness. The Y165F variant, called SumireF, was reported to be a fast-maturing variant of Sumire.^17^ On its own, SumireF is brighter than Sumire due to a higher extinction coefficient and equivalent quantum yield. In combination, superposition of the Y165F mutation with the V206K mutation recovers 60% brightness, and mSumireF is 2-fold brighter than Sirius, the only other violet fluorescent protein.^12^ Neither mutation affects the absorbance, fluorescence excitation, or fluorescence emission spectra significantly (Figure 2 and Figure S2).

**Figure 2:**
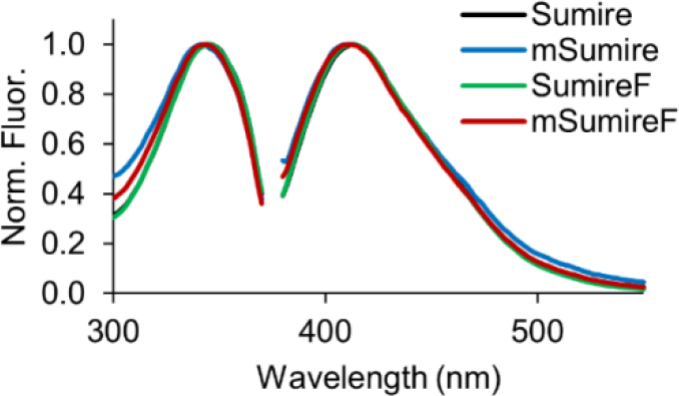
mSumireF Fluorescence Spectra. Excitation (left) and emission (right) for Sumire (black), mSumire (blue), SumireF (green), and mSumireF (red). Spectra are peak normalized.

We additionally measured the practical brightness of Sumire versus mSumireF in mammalian HEK293 cells. HEK293 cells were transfected with Sumire-T2A-mScarlet-I3 or mSumireF-T2A-mScarlet-I3. The viral T2A ribosomal skip sequence ensures one-to-one stoichiometry of the nonfused individual violet and red fluorescent proteins. Relative cell brightness was quantified as the violet-to-red intensity ratio (Figure S3).^15^ Interestingly, Sumire (V/R = 0.31 ± 0.02, mean ± 95% confidence interval, n = 87) expressed in mammalian cells was 2.5-fold brighter than mSumireF (V/R = 0.12 ± 0.01, n= 51). This could be a reflection of both the difference in photophysical brightness as well as a possible difference in maturation efficiency when expressed in mammalian cells. Regardless, both Sumire and mSumireF transfected cells were easily visible in live-cell imaging.

### Reduced Oligomerization

To assess oligomerization tendency in cells, we carried out the organized smooth endoplasmic reticulum (OSER) assay.^7^ p450 was N-terminally fused to Sumire and mSumireF so that fusion proteins expressed in HEK293 cells were localized to the cytosolic face of the SER. In this assay, oligomerization of fluorescent proteins causes puncta and whorls to form (Figure 3).

**Figure 3:**
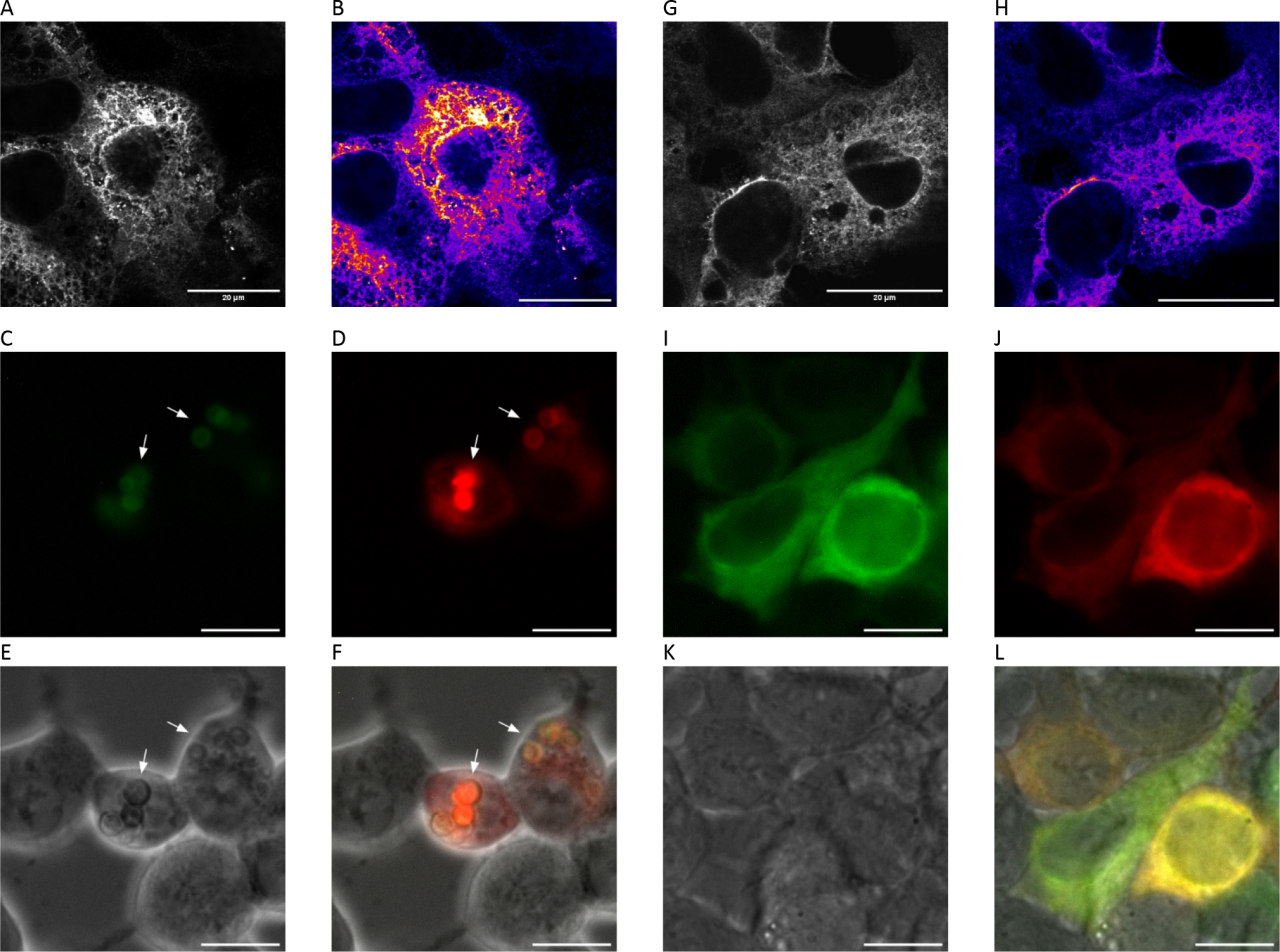
OSER assay shows (A–F) oligomerization of Sumire but (G–L) monomeric behavior of mSumireF. (A,B, G–H) Confocal imaging of fixed and immunostained cells. HEK293 cells expressing (A,B) p450-Sumire on the cytosolic side of the smooth endoplasmic reticulum exhibited bright puncta indicative of oligomerization despite the absence of whorls. (G–H) In contrast, cells expressing p450-mSumireF exhibited largely homogeneous fluorescence across the smooth ER network with an absence of many puncta. (A,G) Grayscale. (B,H) Pseudocolor ranges purple to white to indicate increasing intensity. White pixels indicate saturation for the 16-bit images. Scale bar = 20 μm. (C–F, I–L) Live-cell widefield imaging of HEK293 cells cotransfected with (D,J) DsRed-ER and either (C–F) p450-Sumire or (I-L) p450-mSumireF. (C–F) The majority of p450-Sumire expressing cells exhibited conspicuous whorls (arrows) whereas (I–L) the majority of p450-mSumireF expressing cells showed normal SER. (C,I) violet channel, (D,J) red channel, (E,K) phase contrast, (F,L) overlay. Scale bar 20 μm.

One major drawback of violet fluorescent proteins such as Sumire is that laser ultraviolet (UV) excitation at wavelengths less than 400 nm is no longer commonly available on shared confocal microscopy instruments. Despite this impediment, to image the Sumire variants we fixed and immunolabeled cells with anti-GFP primary and AlexaFluor488 secondary antibodies to observe the expression pattern by confocal microscopy. Immunostained cells exhibited clear reticular fluorescence patterns as evidence that the Sumire variants were correctly targeted to the SER by the p450 fusion (Figure 3). Interestingly, four independent researchers qualitatively identified a stark difference between the immunostained Sumire and mSumireF expressing cells. The staining pattern in mSumireF-expressing cells was largely homogeneous across the SER network within each cell, which appeared healthy and normal. In contrast, the majority of Sumire-expressing cells exhibited bright, saturating puncta among the normal reticular pattern. We suspected that the bright puncta were caused by oligomerization of the wildtype Sumire. We did not observe the typical whorls expected when fluorescent proteins oligomerize in the OSER assay; however, fixation and permeabilization of the cells may have caused artifacts that preclude the observation of pathological whorls. Because of this concern, we also carried out live-cell imaging with a widefield fluorescence microscope equipped with UV excitation for direct observation of the violet fluorescent proteins.

Notably, direct observation of cells expressing p450-mSumireF at high magnification showed localization patterns consistent with the cotransfected DsRed-ER marker, and p450-mSumireF was not mislocalized to the cytosol (Figure 3 and Figures S4 and S5).

Instead, pathological whorls were obvious in almost all p450-Sumire expressing cells (Figure 3 and Figure S4). In contrast, almost all p450-mSumireF expressing cells had normal, reticular SER. We quantified the number of normal cells versus cells exhibiting pathological whorls,^7^ and the OSER score for Sumire was 16 ± 2% normal cells compared to 93 ± 11% normal cells for mSumireF.

We then further extended our analysis to include a quantitative in vitro protein assay. Fluorescence anisotropy is a long established method to measure dimerization, oligomerization, and clustering of fluorescent proteins.^18–21^ We therefore measured oligomerization of the purified Sumire proteins in solution by fluorescence anisotropy, and the quantitative spectroscopic results correlated well with the OSER imaging experiments. Oligomerization of fluorescent proteins alters fluorescence anisotropy by two competing processes. Binding of fluorescent proteins causes an increase in particle size and decrease in rotational rate, which causes an increase in fluorescence anisotropy. However, close proximity of bound fluorescent proteins increases the efficiency of homotypic Förster resonance energy transfer (homoFRET), which depolarizes emission and causes a decrease in fluorescence anisotropy.^22^ Despite a moderate Stokes shift and small overlap between the absorbance and emission spectra, we found that homoFRET can be highly efficient for Sumire and its variants. Therefore, in these experiments a net decrease in fluorescence anisotropy reports binding and oligomerization.

Indeed, increasing the parent Sumire protein concentration caused a drastic decrease in fluorescence anisotropy that was well fit by an empirical Hill equation for binding (Figure 4). The parent Sumire exhibits a high self-affinity with an apparent binding dissociation constant of 2.4 μM (Table 1). Similarly, SumireF, which also lacks the monomerizing mutation, exhibits a high apparent affinity of 3.3 μM. In contrast, mSumire did not exhibit any decrease in anisotropy up to 100 μM protein concentration, suggesting it is well-behaved as a truly monomeric fluorescent protein in solution. Interestingly, mSumireF exhibits significantly less oligomerization tendency and only shows a decrease in anisotropy for high protein concentrations above 50 μM. Although the data for mSumireF could not be well fit, they indicate an apparent self-affinity greater than 80 μM. This is compared to concentrations for heterologous expression of fluorescent proteins in cells, which typically fall in the 0.1 – 10 μM range. Thus, both OSER and fluorescence anisotropy show that Sumire has strong tendency to oligomerize, but mSumireF is a good alternative that has significantly improved monomeric behavior.

**Figure 4:**
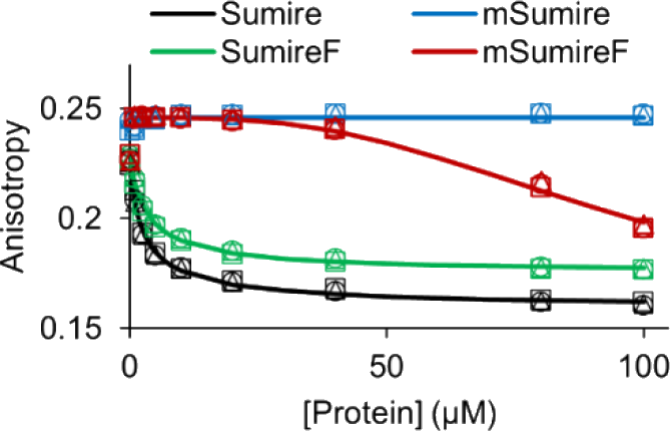
Solution oligomerization assay. Oligomerization increases the efficiency of homoFRET and causes a decrease in fluorescence anisotropy for Sumire (black) and SumireF (green). mSumire (blue) does not exhibit oligomerization at all, and mSumireF (red) exhibits significantly reduced oligomerization tendency. Symbols are individual replicates (*n* = 3). Lines are fitted binding curves using an empirical Hill equation.

### Live Cell Spectroscopy

Next, we were interested in possible applications of mSumireF. In particular, fluorescent proteins and probes are used extensively to study bacteria^23,24^ and can be of great use in nonimaging spectroscopic modalities.^25^ However, one major concern for the use of violet fluorescent proteins expressed in bacterial suspensions is that UV excitation can elicit high background autofluorescence in addition to scatter. As such, we asked whether mSumireF fluorescence could be detected above background in *E. coli* suspension cultures using steady-state fluorescence measurements in a plate reader. Furthermore, the Y165F mutation was reported to increase the maturation rate of SumireF,^17^ and so we also asked how soon after induction could we detect the onset of fluorescence for all four Sumire variants. As a comparison, each Sumire variant was fused to mScarlet-I3.^15^ mScarlet-I3 has one of the fastest maturation times of 2 min, providing a spectrally compatible red fluorescent reference.

Overall, violet fluorescence could be detected above background for all four Sumire variants within 1.5 h after induction of expression, and none of the variants qualitatively impeded mScarlet-I3 expression (Figure 5). We were careful to use nonfluorescent control bacteria to subtract background autofluorescence, and we found that violet and red fluorescence onset was consistently linear and reproducible at early times after induction. As mentioned, we used a long 27 amino acid linker to fuse mScarlet-I3 to each Sumire variant so that it would be possible for the two fluorescent proteins to fold relatively independently. However, it is possible that the fast, efficient folding of mScarlet-I3 affected the folding of the Sumire variants. The onset of violet fluorescence may be slower in other contexts, but the key observation here is that mSumireF is bright enough for robust detection above background even in bacterial suspension cultures. Thus, mSumireF expands the color palette for multiplexed live-cell spectroscopy experiments.

**Figure 5:**
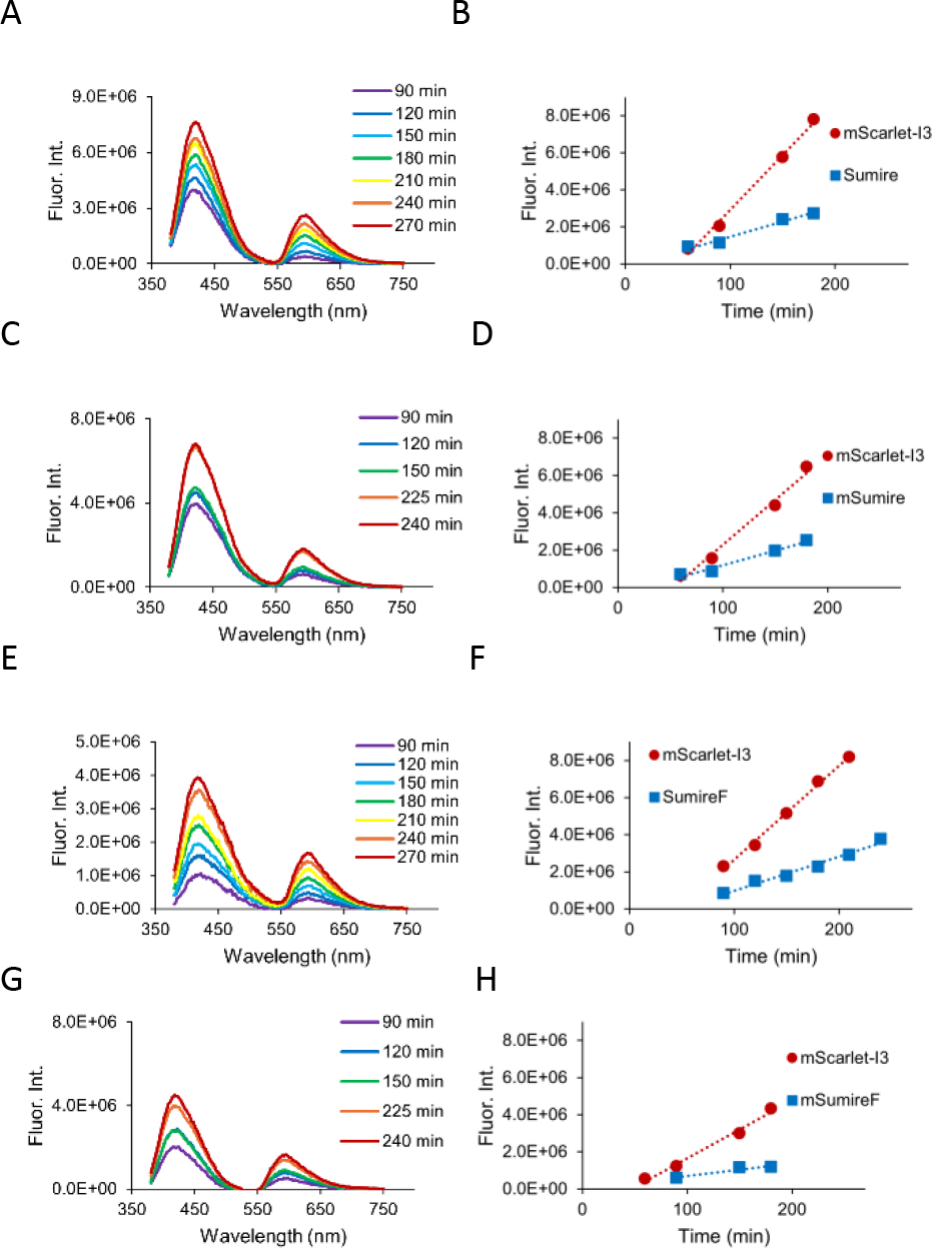
Spectroscopic detection in live bacterial cultures. The onset of fluorescence above background in *E. coli* suspensions was measured relative to mScarlet-I3 fused to (A,B) Sumire, (C,D) mSumire, (E,F) SumireF, and (G,H) mSumireF. (A,C,E,G) Background subtracted fluorescence emission spectra are shown as a rainbow-colored series overexpression time. The Sumire emission peak is at 415 nm. Spectra show bleed-through excitation of mScarlet-I3 that results in the secondary peak at 590 nm. (B,D,F,H) Onset of violet fluorescence for the Sumire variant (blue) compared to the fused mScarlet-I3 reference (red). mScarlet-I3 fluorescence intensities were measured using direct excitation of mScarlet-I3.

### FRET

Finally, we demonstrated that mSumireF can serve as a traditional heterotypic FRET donor to various different acceptor fluorescent proteins. Sugiura and Nagai originally demonstrated that the parent Sumire and T-Sapphire have significant spectral overlap and act as an effective FRET pair in the context of a calmodulin-M13 Cameleon-type calcium FRET sensor.^2,26^ Based on spectral overlap, we selected four additional candidate fluorescent protein acceptors to test in the context of an ATeam-type ATP FRET sensor (Figure 6).^27,28^ Of the four, mTagBFP2 ^29^ did not exhibit significant FRET with mSumireF and was not further pursued.

**Figure 6:**
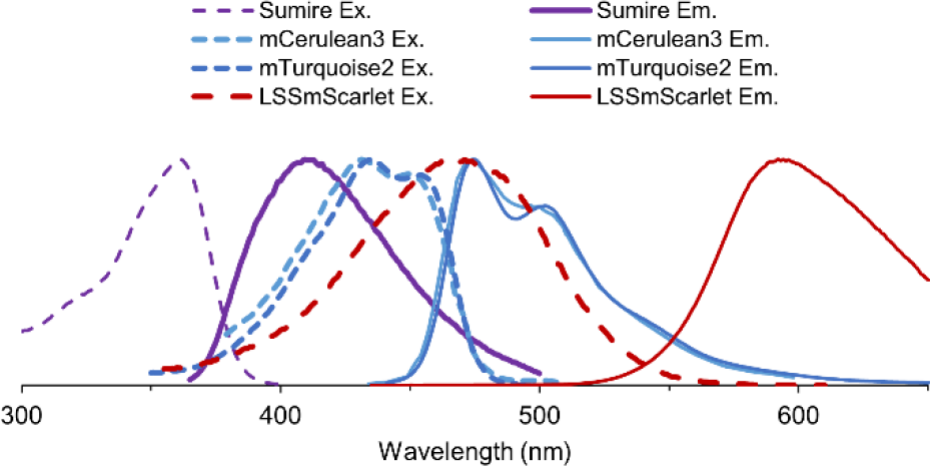
Spectral overlap between Sumire and potential FRET acceptors. Spectra generated using FPbase.^27^

The remaining three candidates, mCerulean3,^30^ mTurquoise2,^31^ and LSSmScarlet^32^ all exhibited significant FRET capability with an mSumireF donor. In the ATeam-scaffolded sensors, the donor and acceptor fluorescent proteins are fused to the N- and C-termini of the *B. subtilis* ATP synthase epsilon subunit. In the absence of ATP, the N-terminal β sandwich and C-terminal α helix adopt a flexible extended conformation in which the donor and acceptor fluorescent proteins are far from one another making FRET inefficient.^28,33^ ATP binding induces a conformational change in which the C-terminal domain folds into an ATP-bound helix-turn-helix conformation that brings the acceptor and donor fluorescent proteins close together and increases FRET efficiency. In this ATP sensor context, mSumireF paired with mCerulean3, mTurquoise2, and LSSmScarlet all exhibited ATP does-response curves at physiological cytosolic ATP concentrations with affinity comparable to the mseCFP-mVenus ATeam parent, though with reduced dynamic range (Table 2 and Figure 7). Future optimization of linker lengths or use of circularly permuted variants could improve the dynamic ranges, but even so, our proof-of-concept illustrates that mSumireF can expand the color palette of ATeam sensors for the measurement of physiological ATP concentrations in live cells. This may be particularly useful given the increasing prevalence of multiplexed experiments using two or more biosensors that must be color compatible.

**Table 2:** FRET and ATP Sensor Properties

	ε^a^	φ^b^	J^c^	R_0_ ^d^	ΔF/F_0_ ^e^	K_D_,_app_ ^f^
mCerulean3	40	0.87	0.86	47.34	22 ± 6%	2.3 ± 0.6
mTurquoise2	30	0.93	0.61	44.72	19 ± 1%	3 ± 1
LSSmScarlet	30	0.42	0.56	44.08	23 ± 7%	3 ± 1

a Acceptor extinction coefficient mM^–1^·cm^–1^.

b Acceptor quantum yield.

c Overlap integral x 10^–15^ M^–1^·cm^–1^·nm^4^.

d Förster radius Å.

e ATP sensor percent change dynamic range.

f ATP sensor apparent affinity; ε, φ, J, R_0_ from FPbase.^27,34^

**Figure 7:**
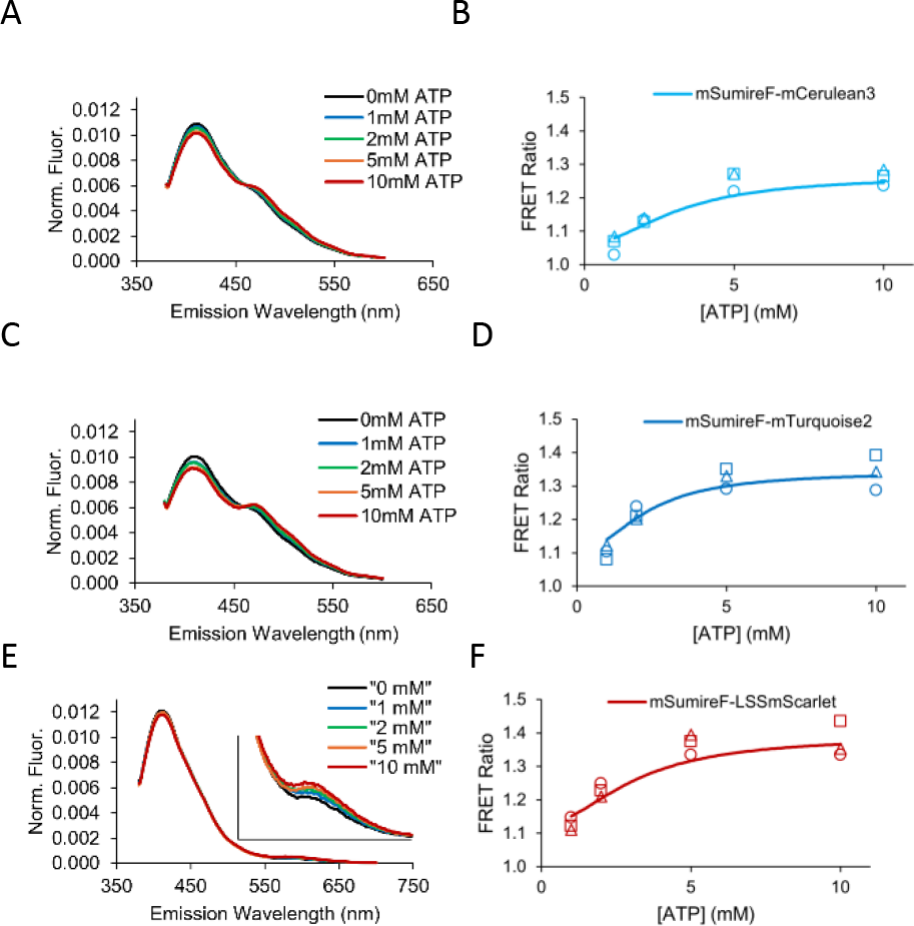
mSumireF FRET-based ATP Sensors. ATeam-type sensors utilizing mSumireF paired with (A,B) mCerulean3, (C,D) mTurquoise2, and (E,F) LSSmScarlet quantitatively detect different concentrations of ATP in solution, demonstrating three possible FRET acceptors that can be paired with an mSumireF donor. (A,C,E) Spectral changes with increasing ATP concentration. (B,D,F) ATP dose–response curves.

Interestingly, we found that these three mSumireF-based FRET sensors can also report ATP changes via sensitized acceptor emission anisotropy. Traditionally, two-color FRET sensors report analyte changes by way of a ratiometric spectral change. Recently, it was demonstrated that the depolarization of acceptor emission caused by FRET can be measured as an alternative readout.^35^ Upon excitation of the donor, acceptor emission is the sum of FRET intensity and fluorescence from bleed-through acceptor excitation. Bleed-through acceptor fluorescence has high anisotropy due to slow rotation of the fluorescent protein whereas the process of FRET depolarizes the emission angle so that sensitized acceptor emission has low anisotropy. Indeed, we found that ATP addition consistently decreases sensitized acceptor emission anisotropy by 0.02 to 0.03 (Figure S6). Notably, assays were carried out using 0.5 μM protein so that the very weak oligomerization tendency of mSumireF was not a concern. Thus, mSumireF has the capacity to generate at least three new color options for ATP detection in at least two different FRET detection modalities.

## Conclusions

We report that mSumireF, the Sumire­(V206K/Y165F) mutant, is a bright, monomeric violet fluorescent protein. We found that the parent Sumire exhibits a low OSER score of 16% in mammalian cells with conspicuous oligomerization pathologies apparent using phase contrast even without fluorescence. It also has a surprisingly high self-affinity with apparent dissociation constant less than 10 μM, whereas Zacharias and Tsien and co-workers originally reported a dissociation constant of approximately 100 μM for the “non-monomeric” GFP-derived YFP.^16^ We suspect that the N146I mutation found in Sumire, relative to its sfGFP parent, may contribute to the increased dimerization we observed for protein in solution and in cells. N146 is found on a β-strand neighboring V206 in the GFP dimer interface, and therefore substitution with a hydrophobic isoleucine could increase the driving force for dimer formation.^36,37^ This high propensity for dimerization presents a serious drawback for the use of Sumire, and therefore we carried out mutagenesis to monomerize it.

We found that the canonical V206K monomerizing mutation eliminates oligomerization tendency *in vitro*. Unfortunately, this monomerization comes at the expense of brightness. The canonical 206K monomerizing mutation for GFP-family fluorescent proteins often does not affect photophysical brightness,^38^ but loss of brightness upon monomerization has in fact been observed for several other fluorescent proteins.^16,39–41^ In addition, it has been observed that external facing residues can strongly affect chromophore photophysics in some cases.^42^ However, through additional mutagenesis, we found that combining the Y165F mutation with V206K recovers the majority of brightness and maintains reduced oligomerization tendency as observed with an OSER score of 93% in mammalian cells. Interestingly, the original parent Sumire included the F165Y mutation relative to sfGFP to improve brightness. In contrast, here in the context of the V206K monomer, reversion of Y165 back to F recovers brightness. Residue 165 has been reported to contribute to electron transport pathways, lifetime, and quantum yield,^43,44^ thus its location close to the dimer interface may couple its role to oligomerization state. For example, mSumire exhibits no oligomerization tendency at all whereas mSumireF exhibits very weak oligomerization tendency in the anisotropy assay. This observation is interesting because residue 165 is internal facing, suggesting that the Y165F mutation may alter folding or internal packing in such a way that the structural changes affect the dimer interface. Conversely, oligomerization could also cause small structural changes to the internal packing that affect position 165 and the chromophore environment.^45^

We also demonstrated that the mSumireF variant is bright enough for detection above background autofluorescence and scatter in live bacterial suspensions. When fused to mScarlet-I3 as a reference, the onset of violet fluorescence was evident as early as 90 min after induction, and thus mSumireF is an important addition to the microbial fluorescent protein toolkit. Interestingly, when expressed in mammalian cells with mScarlet-I3 as a reference, mSumireF is easily visible but 2.5-fold dimmer than the parent Sumire. Relative cell brightness takes into account folding and maturation efficiency as well as photophysical brightness. Given the brightness of the mSumireF purified protein, these results suggest that folding and maturation in mammalian cells may not be as efficient as the Sumire parent. It is also possible that dimerization of the original Sumire improves its cell brightness, which is then lost with the monomeric mSumireF. Despite the lower relative mammalian cell brightness, the monomeric nature of mSumireF is a critical improvement over Sumire.

Finally, to demonstrate one application in which mSumireF can provide expanded color options, we showed that it can serve as a traditional FRET donor to two different cyan fluorescent proteins and a long Stokes Shift red fluorescent protein. We report three different color prototypes for ATP sensors as a proof-of-principle, and in the future these prototypes could be optimized to improve dynamic range or modify ATP affinity as needed.

Overall, our results show that mSumireF is an improved violet fluorescent protein that contributes to the expanded spectral range of monomeric fluorescent proteins.

## Supplementary Material



## Notes

Quantum yield; absorbance spectra; relative cell brightness; OSER assay images; sensitized acceptor fluorescence anisotropy (PDF)
