# Supplementary material for: mSumireF a Monomeric Violet Fluorescent Protein
Source: ACS Bio Med Chem Au. 2026 Jan 6;6(1):36–43. doi: 10.1021/acsbiomedchemau.5c00175 (PMC12921512; doi:10.1021/acsbiomedchemau.5c00175)
Supplement: Supplementary file 1 [file bg5c00175_si_001.pdf]

SUPPORTING INFORMATION

mSumireF a Monomeric Violet Fluorescent Protein

Jacob Kress, Sookyeong Kim, Caitlynn Bryant, Emily Camila Lopez-Lopez, Jiali Zha, Mathew Tantama*

Department of Chemistry & Biochemistry Program, Wellesley College, 106 Central Street, Wellesley, MA 02481.

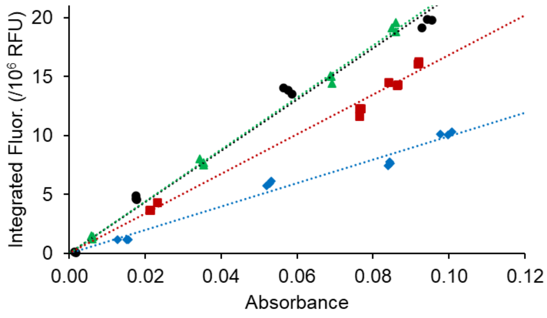

Figure S1. Fluorescence quantum yield determination from integrated fluorescence vs. absorbance plots. Triplicate data sets were individually well fit to linear functions for Sumire (black, average R^2 = 0.994), mSumire (blue, average R^2 = 0.994), SumireF (green, average R^2 = 0.999), and mSumireF (red, average R^2 = 0.998).

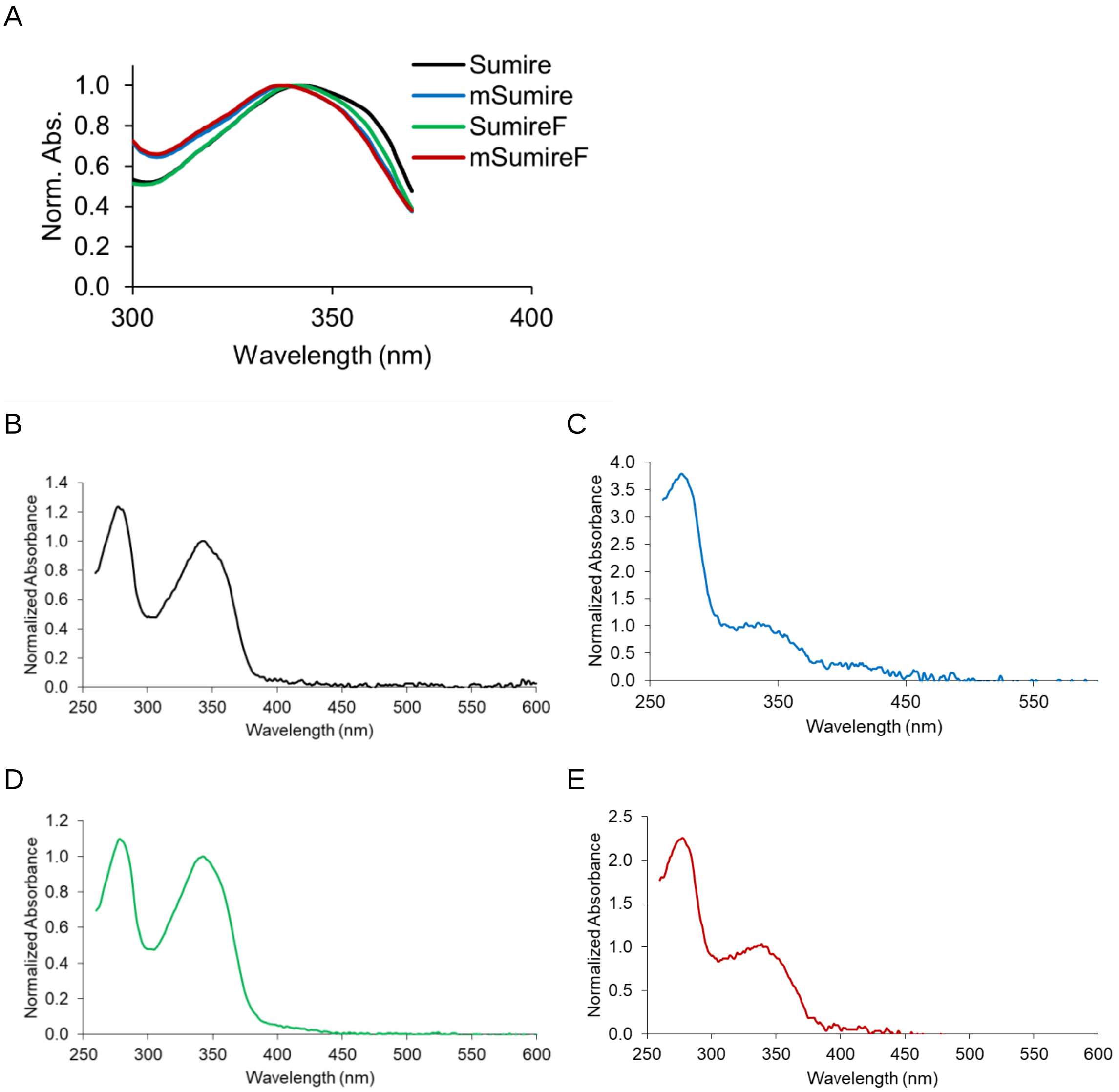

Figure S2. Absorbance spectra. (A) Absorbance spectra were collected over the same wavelength range as fluorescence excitation spectra, starting at 300 nm to exclude the canonical 280 nm peak for all proteins. Full absorbance spectra measured from 260 nm to 600 nm for (B) Sumire, (C) mSumire, (D), SumireF, and (E) mSumireF.

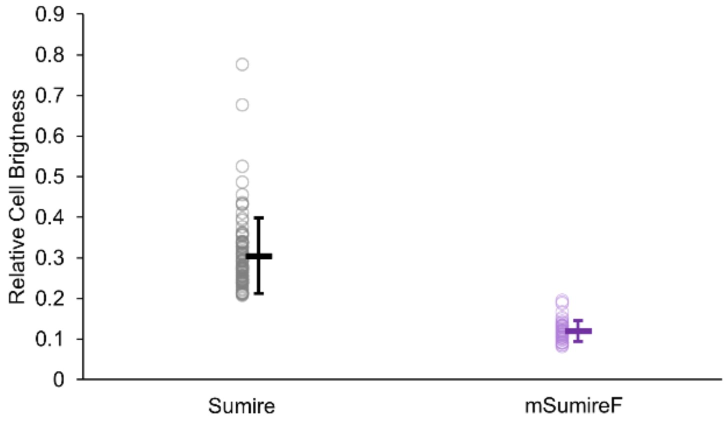

Figure S3. Relative cell brightness. HEK293 cells were transfected with Sumire-T2A-mScarlet-I3 (n = 87 cells) or mSumireF-T2A-mScarlet-I3 (n = 51 cells). Relative cell brightness was quantified as the ratio of violet-to-red (V/R) integrated intensities using the same instrumental settings for all images of the same color channel. The plot shows individual cell V/R ratios, and the mean with standard deviation as error bars is shown offset. A Nikon ET-DAPI (96360) filter cube was used for violet fluorescence, and a Nikon ET-DsRed (96364) filter cube with an OD8 neutral density filter was used for red fluorescence. Integration times were held constant at 500 ms. Thresholds were set to a minimum of three-times background and cells with saturated pixels were excluded. Widefield imaging was carried out on a Nikon Ti-U inverted microscope with metal halide lamp illumination, a QI Click Monochrome CCD camera, and 40X 0.6 NA CFI Super Plan Fluor air objective. Cells were imaged in DPBS supplemented with 10 mM glucose.

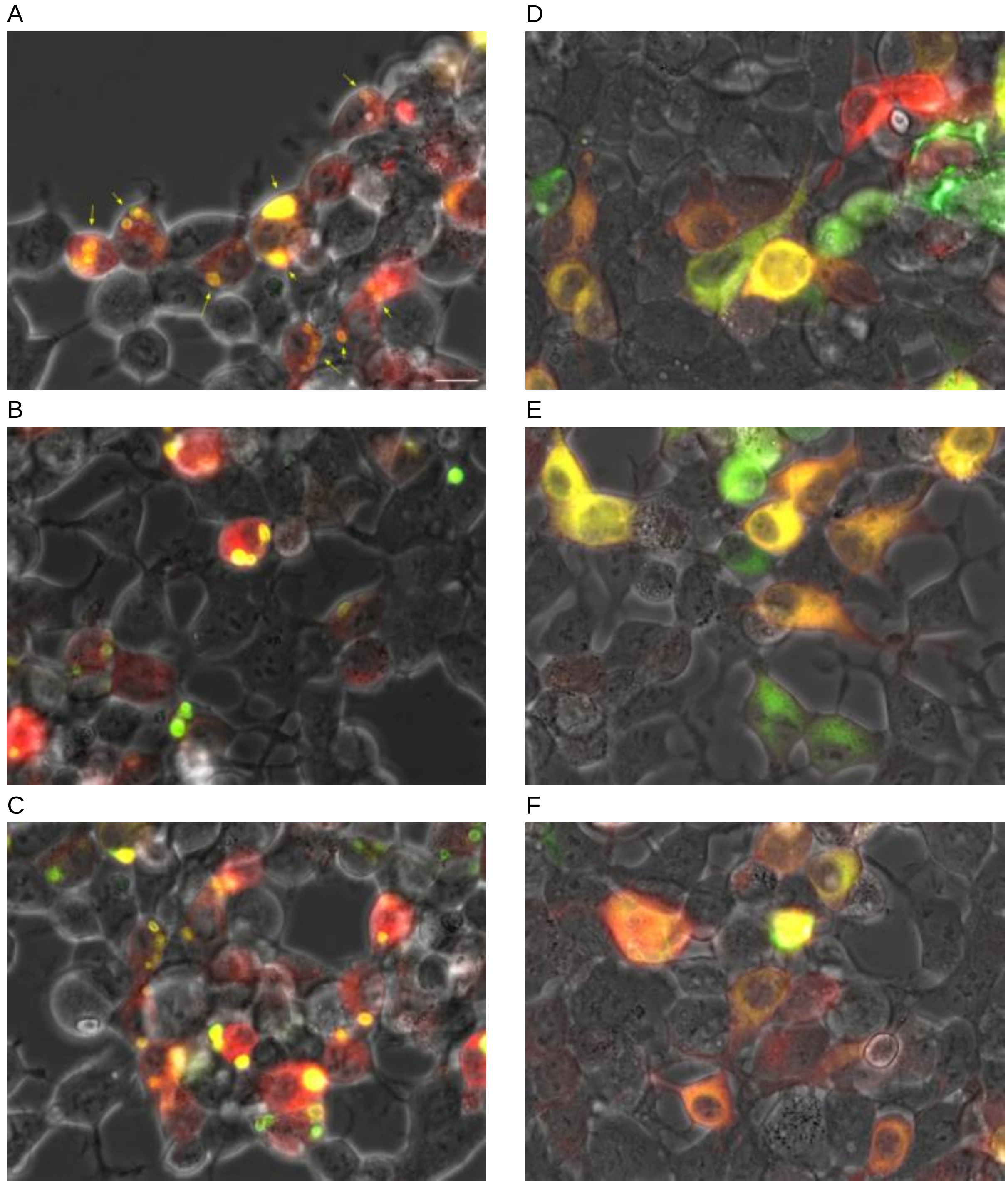

Figure S4. OSER Assay with widefield imaging. HEK293 cells were co-transfected with DsRed2-ER and (A-C) p450-Sumire or (D-F) p450-mSumireF. (A) p450-Sumire expressing cells show highly conspicuous whorls (arrows) in the majority of transfected cells. Scale bar 20 µm. Overlay of phase contrast (grey), DsRed2-ER (red), and p450-Sumire (green). Fluorescence intensity contrast was autoscaled in ImageJ. Representative images for (A-C) p450-Sumire expressing cells with whorls versus (D-F) p450-mSumireF expressing cells that conspicuously lacked pathological whorls. Widefield imaging was carried out as described for Figure S3.

A

B

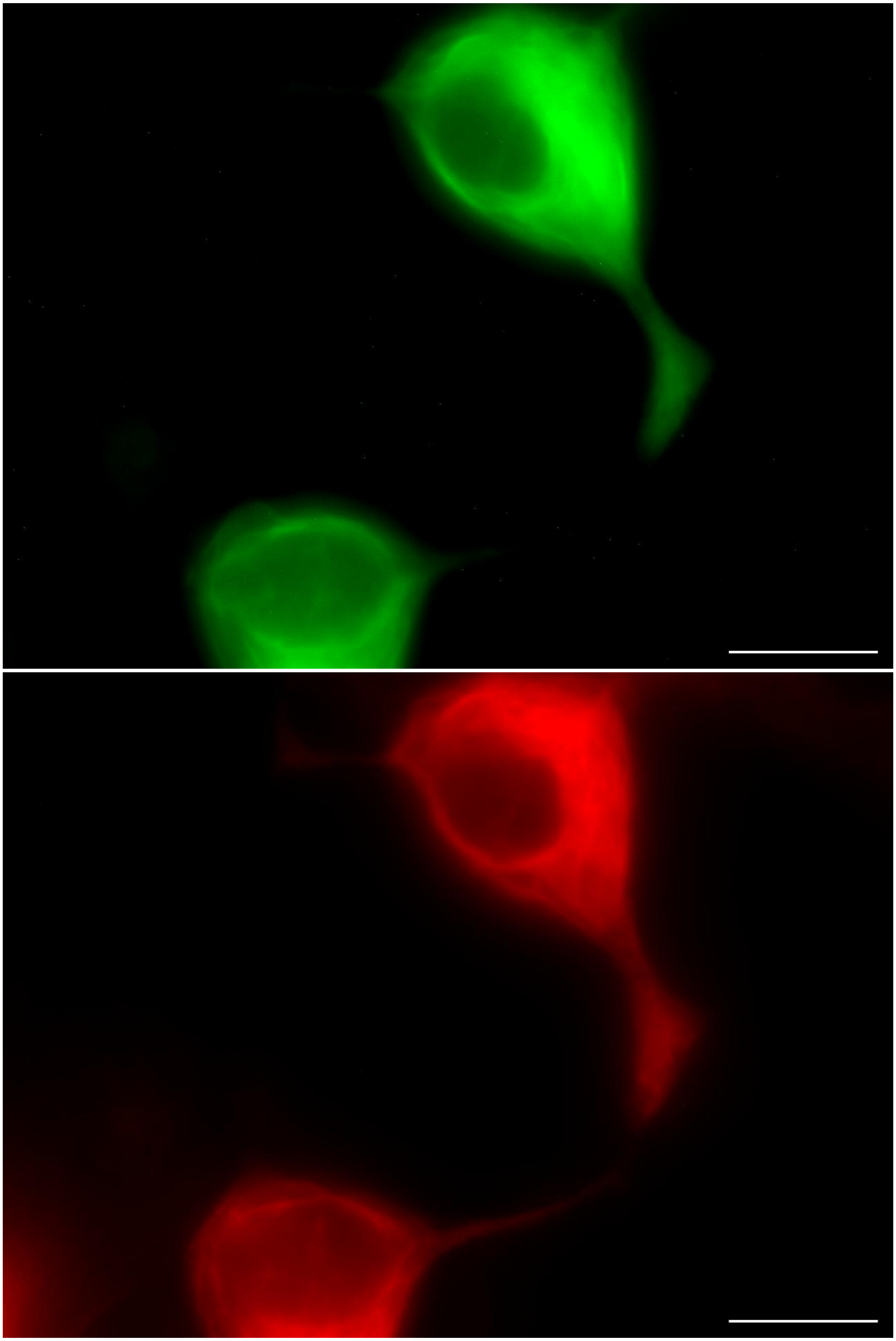

C

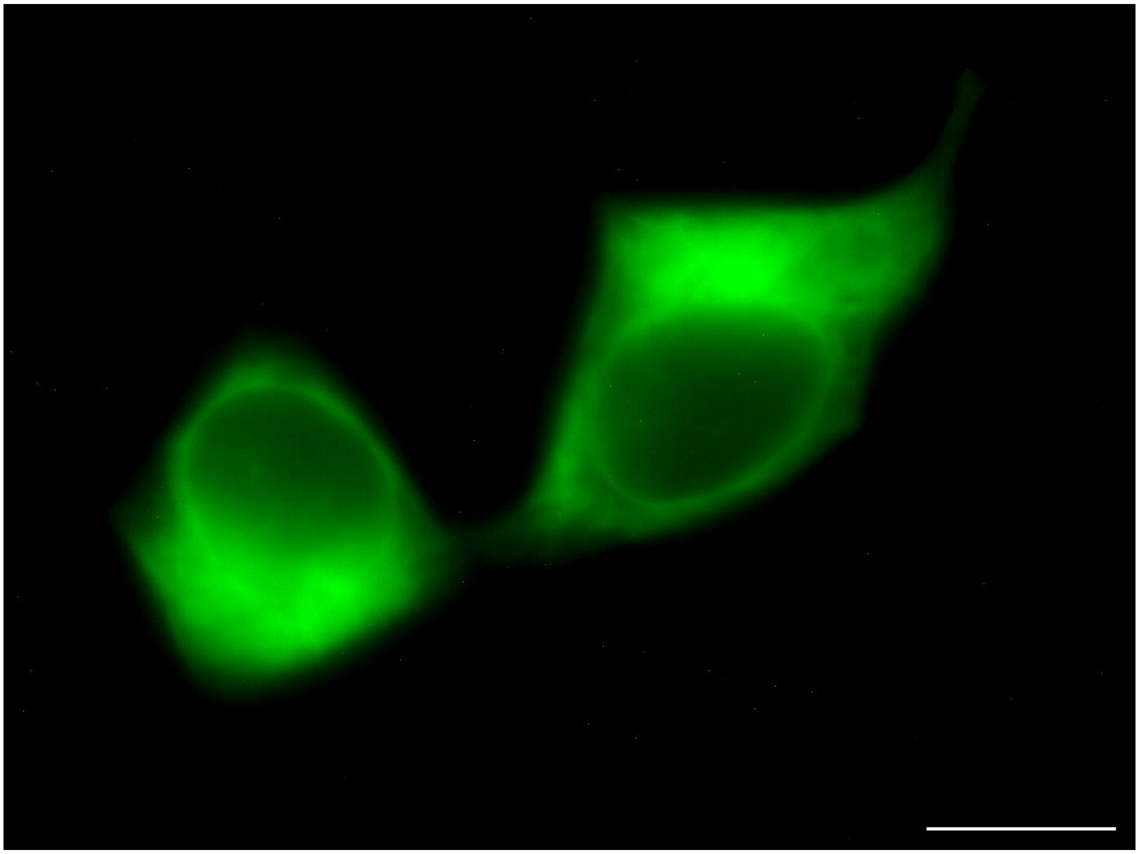

D

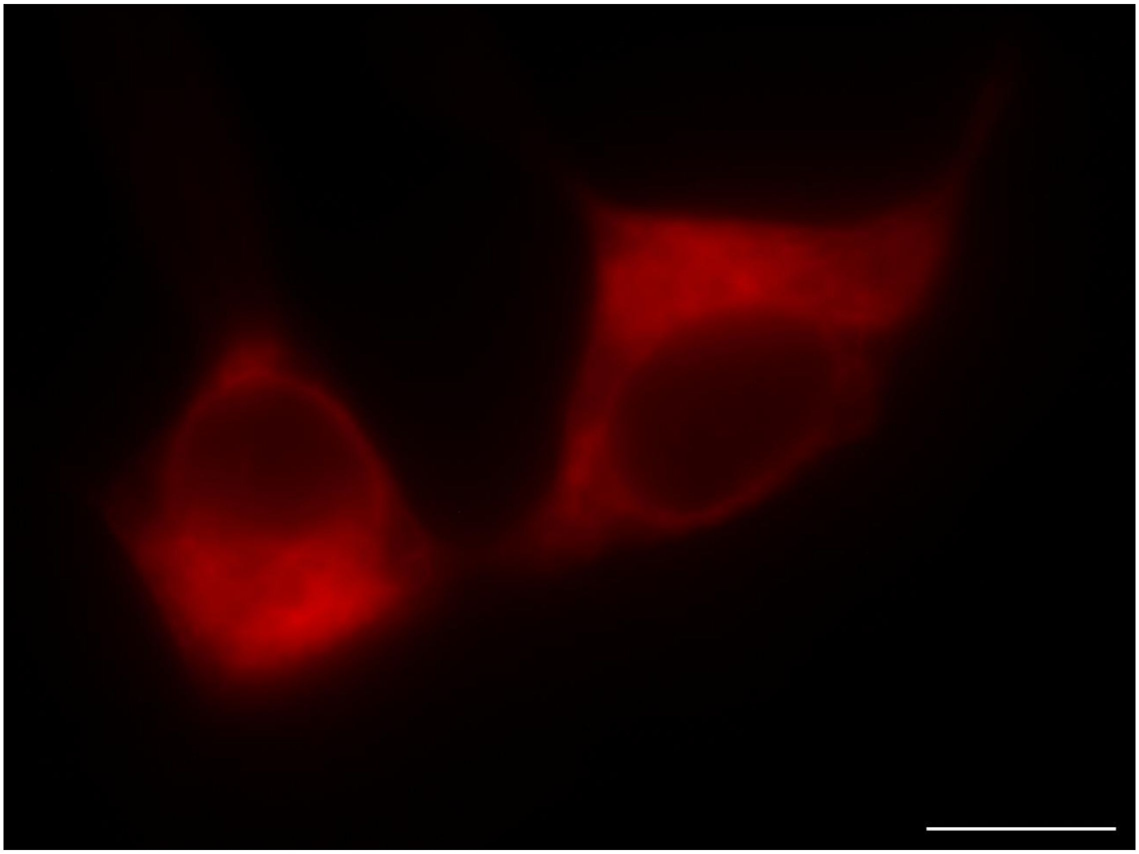

Figure S5. Evidence of correct subcellular localization for p450-mSumireF compared to the DsRed2-ER marker in the OSER assay. HEK293 cells were co-transfected with p450-mSumireF and DsRed2-ER just as in Figure 3 and S4. Unbinned widefield images were taken with a Nikon 100X 1.30 NA Oil Plan Fluor objective with an additional 1.5X intermediate magnification from the Nikon Ti-U microscope. (A,C) Images of p450-mSumireF were taken in sequence first, and then subsequently (B,D) images of DsRed2-ER for the same field of view were taken in sequence. 20 frames were averaged and autocontrast was applied in FIJI/ImageJ. Scale bars are 10 μm. Because of manual operation, imaging of each channel in the field of view took several minutes during which cell movement was apparent between channels. Despite this, comparison of p450-mSumire versus DsRed2-ER localization patterns were consistent with proper ER localization and no apparent mislocalization to the cystosol.

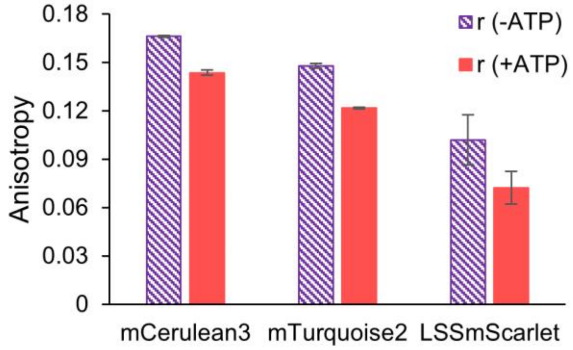

Figure S6. Sensitized acceptor fluorescence anisotropy. Anisotropy was measured by exciting mSumireF and recording sensitized acceptor emission.
